# A Voyage on the Role of Nuclear Factor Kappa B (NF-kB) Signaling Pathway in Duchenne Muscular Dystrophy: An Inherited Muscle Disorder

**DOI:** 10.7759/cureus.67901

**Published:** 2024-08-27

**Authors:** Akshaya R, Sumithra Mohan, Chitra Vellapandian

**Affiliations:** 1 Pharmacy/Pharmacology, Sri Ramaswamy Memorial College of Pharmacy, Sri Ramaswamy Memorial Institute of Science and Technology, Kattankulathur, IND; 2 Pharmacology, Sri Ramaswamy Memorial Institute of Science and Technology, Kattankulathur, IND

**Keywords:** glucocorticoids, fibrosis, inflammation, muscle degeneration, dystrophin, nf-κb signaling pathway, duchenne muscular dystrophy (dmd)

## Abstract

A recessive X-linked illness called Duchenne muscular dystrophy (DMD) is characterized by increasing muscle weakening and degradation. It primarily affects boys and is one of the most prevalent and severe forms of muscular dystrophy. Mutations in the *DMD* gene, which codes for the essential protein dystrophin, which aids in maintaining the stability of muscle cell membranes during contraction, are the cause of the illness. Dystrophin deficiency or malfunction damages muscle cells, resulting in persistent inflammation and progressive loss of muscular mass. The pathophysiology and genetic foundation of DMD are thoroughly examined in this review paper, focusing on the function of the NF-κB signaling system in the disease’s progression. An important immune response regulator, NF-κB, is aberrantly activated in DMD, which exacerbates the inflammatory milieu in dystrophic muscles. Muscle injury and fibrosis are exacerbated and muscle regeneration is hampered by the pro-inflammatory cytokines and chemokines that are produced when NF-κB is persistently activated in muscle cells. The paper also examines our existing knowledge of treatment approaches meant to inhibit the progression of disease by modifying NF-κB signaling. These include new molecular techniques, gene treatments, and pharmacological inhibitors that are intended to lessen inflammation and improve muscle healing. Furthermore covered in the analysis is the significance of supportive care for DMD patients, including physical therapy and corticosteroid treatment, in symptom management and quality of life enhancement. The article seeks to provide a thorough understanding of the mechanisms causing DMD, possible therapeutic targets, and developing treatment options by combining recent research findings. This will provide clinicians and researchers involved in DMD care and research with invaluable insights.

## Introduction and background

**Figure 1:**
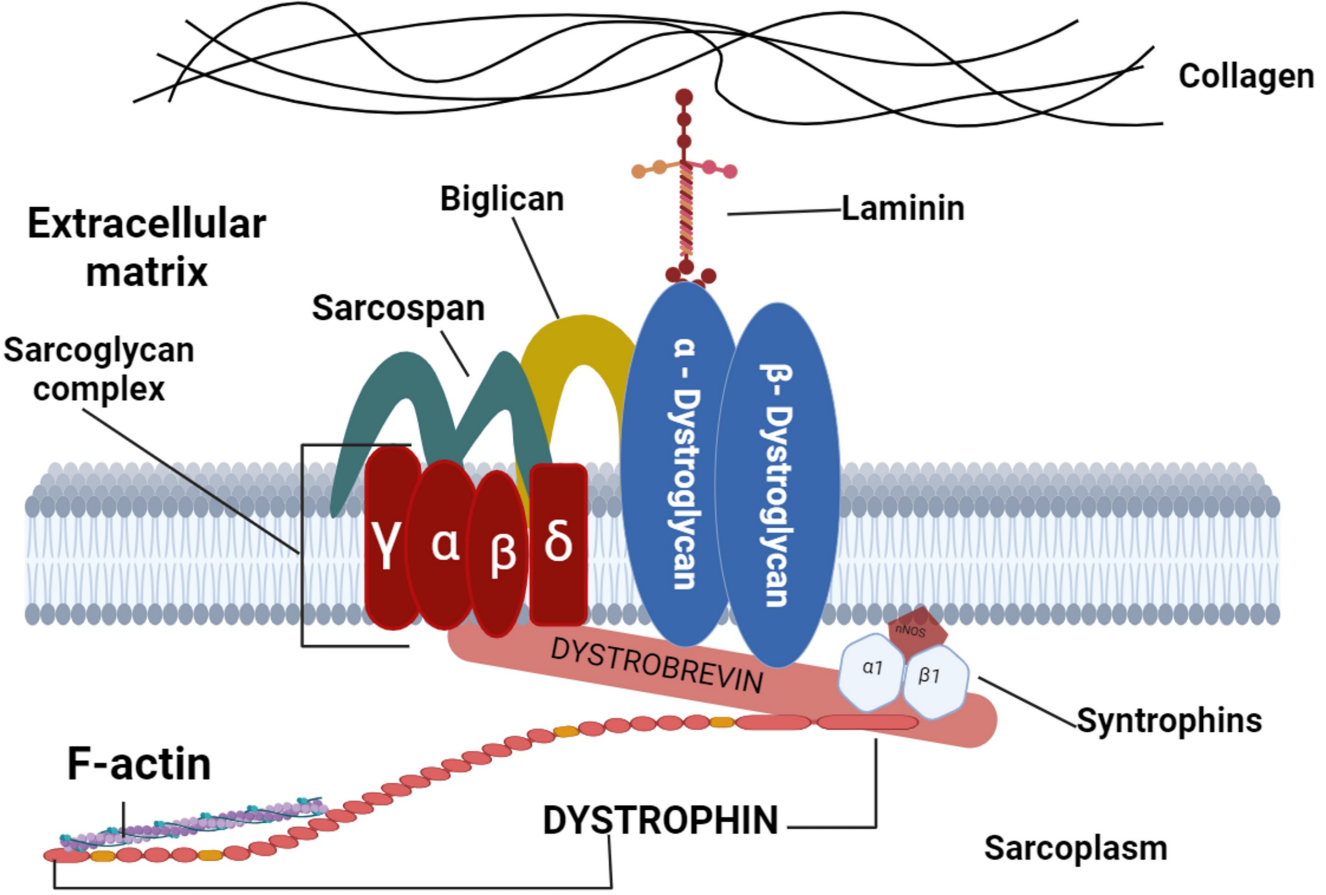
This figure depicts the dystrophin-glycoprotein complex (DGC). DGC connects muscle cell cytoskeletons to the extracellular matrix, providing structural stability to muscle fibers. Key components are: Collagen and laminin form the extracellular matrix, which provides structural support. Sarcospan and the sarcoglycan complex (α, β, γ, δ) are transmembrane proteins that maintain muscle integrity. Dystroglycans (α and β) connect the extracellular matrix to the cell membrane. Intracellular proteins: Dystrophin connects actin filaments to the sarcolemma, while dystrobrevin and syntrophins help with signaling and structural stability. Original illustration by the author.

Duchenne muscular dystrophy (DMD) is a serious and progressive genetic disorder caused by mutations in the *DMD* gene. These mutations lead to the absence of a functional dystrophin protein, essential for muscle function. Without this critical protein, individuals with DMD experience gradual muscle degeneration and weakening over time. The absence of functioning dystrophin causes substantial damage to muscular strength and function, emphasizing the protein’s critical involvement in muscle integrity and health. As a result, DMD significantly impacts the quality of life and mobility of affected individuals, making it a devastating and challenging condition to manage [1]. Without treatment, boys with DMD typically become wheelchair-dependent by the age of 12. The progression of this debilitating disease continues to affect various muscle groups, and without effective intervention, it often results in severe complications. Tragically, many individuals with DMD succumb to the disease in their late teens, primarily due to respiratory or cardiac failure, underscoring the critical need for early diagnosis and management to potentially improve quality of life and extend life expectancy [2]. There is presently no cure for DMD; however, researchers are actively pursuing a solution. Existing treatments attempt to halt or delay the progression of the disease, offering some hope for those affected. The *DMD* gene (Figure 1), recognized as the largest human gene, consists of 79 exons. It encodes a protein called dystrophin, which is composed of 3,685 amino acids. This protein is crucial for maintaining muscle function and integrity, and its absence or deficiency causes severe symptoms associated with this disorder. Dystrophin is an important cytoskeletal protein found on the inner side of muscle cell membranes. This link is essential for keeping muscle cells structurally intact and stable during contraction and relaxation. By linking the dystrophin-associated protein complex (DAPC) to the actin cytoskeleton, dystrophin helps to protect muscle fibers from damage caused by mechanical stress, thereby playing a crucial role in muscle function and resilience [3].

The NF-κB pathway activates pro-inflammatory chemicals, including adhesion molecules, chemokines, and cytokines, which are important for immune responses and tissue repair. In the context of muscle atrophy, NF-κB activation is essential for mediating the complete process. It contributes to the breakdown of muscle proteins and the inhibition of protein synthesis, ultimately leading to the loss of muscle mass and function seen in conditions like DMD and other muscle-wasting disorders [4]. The NF-κB protein complex regulates DNA transcription, cytokine production, and cell survival. It serves as a pivotal regulator of the immune response to infection, orchestrating the expression of genes that control inflammation and immune reactions. By activating the transcription of various target genes, NF-κB ensures an effective response to pathogens and other stressors, making it essential for maintaining immune system function and cellular health [5]. One important treatment approach for avoiding muscle atrophy is the reduction of NF-κB activation. In addition to its role in controlling inflammation, NF-κB is crucial for various other cellular processes. NF-κB also orchestrates immune system responses to infections by activating genes that encode cytokines, chemokines, and cell adhesion molecules essential for immune cell recruitment and function. Furthermore, NF-κB itself regulates its own pathway, ensuring a controlled and balanced immune response. Thus, NF-κB serves as a central mediator in coordinating cellular responses to stress, infection, and inflammation, highlighting its multifaceted role in maintaining homeostasis and defending against pathogens.

An extensive summary of the state of knowledge about DMD is what this review article aims to deliver, and the NF-κB signaling pathway plays a crucial part in the mechanism of this disease. The review seeks to explain the pathophysiological and genetic causes of DMD, as well as the NF-κB signaling pathway’s activation and dysregulation, which contributes to illness progression. Additionally, it explores therapeutic strategies targeting NF-κB signaling and highlights patient perspectives and supportive care measures crucial for managing the disease. Through this, the article seeks to elucidate potential therapeutic targets and improve management strategies for DMD.

## Review

Genetics and pathophysiology of Duchenne muscular dystrophy (DMD)

DMD is caused by X-chromosomal *DMD* gene mutations. The *DMD* gene produces dystrophin, a protein necessary for muscle fiber integrity and function. This gene is the largest in the human genome, containing 79 exons. Mutations cause dystrophin to be absent or malfunctioning, resulting in DMD symptoms [5]. There are different types of mutation which are Large Deletions - this is the most common type of mutation that occurs in 60-70% of DMD cases, Large Duplications - 5-10% of DMD cases account for this mutation, and Small Mutations - small insertions or deletions, and splice site mutations are responsible for the remaining 25-35% of DMD cases [6]. These mutations lead to a decrease in the amount of functional dystrophin proteins which then causes muscle fiber damage and muscle weakness. Mutations in DMD (encoding dystrophin) prevent the production of the muscle isoform of dystrophin (Dp427m). Dytrophin’s N-terminal and C-terminal domains in muscle connect cytoskeletal F-actin with the extracellular matrix. In DMD, frameshifting or nonsense mutations cause protein translation to be prematurely truncated, resulting in non-functional and unstable dystrophin. Nonsense-mediated decay does not appear to influence these dystrophin transcripts, although epigenetic modifications cause a decrease in transcript synthesis [7]. Without dystrophin, the muscle cell membrane (sarcolemma) becomes fragile, leading to repeated cycles of damage and repair, which ultimately results in cell death. This results in intrinsic myofibrillar necrosis, inflammation, and oxidative stress [8]. Progressive muscle weakness first affects the proximal lower limb and trunk muscles, then the upper limb and distal muscles. Cardiomyopathy, restrictive lung disease, and respiratory failure are also caused by muscle damage to the heart and respiratory systems [2]. *DMD* gene deletions and duplications can be found using the diagnostic technique known as multiplex ligation-dependent probe amplification (MLPA). To find minor alterations, screening techniques like denaturing gradient gel electrophoresis (DGGE) and denaturing high-performance liquid chromatography (DHPLC) are utilized. The mutation in the *DMD* gene is very important for developing targeted therapies [9].

Mechanical stress causes muscular injury in healthy muscles, which triggers inflammation and the recruitment of immune cells to the areas of damage. When activated, recruited immune cells proliferate and produce chemokines and cytokines that cause a local inflammatory response. This, together with the increased oxidative stress at the location of muscle injury, draws more effector immune cells. Simultaneously, innate immune cells aid in tissue regeneration by stimulating the proliferation and maturation of satellite cells, which are progenitor cells of myofibers [10]. In DMD, the improper control of regeneration processes leads to the increasing of inflammation, the activation of the immune system, and the subsequent invasion of cytokines [11]. These cytokines include CD4+ and CD8+ T cells, dendritic cells, B cells, neutrophils, and macrophages with pro-inflammatory M1 and tissue regeneration-focused M2 phenotypes. CD4+ T cells contribute to muscle fiber loss by supplying inflammatory cytokines to CD8+ T cells and other immune cells [12]. The high production of reactive oxygen species (ROS) causes the activation of apoptotic death regulatory proteins due to DNA damage and the breakdown of nuclear and mitochondrial membranes [13]. Activation of nuclear factor-kappaB (NF-κB), oxidative stress, and pro-inflammatory cytokines (e.g., TNF-α, IL-1ß, and TGF-ß) appear to be inflammatory-mediated processes integrated into the progression of muscular dystrophy [14]. Patients with DMD are diagnosed at the age of four, but many boys have symptoms sooner owing to proximal muscular weakness, which causes delayed physical milestones (e.g., walking, running, and climbing stairs). As the condition advances, patients typically become non-ambulatory in their early teens, followed by a gradual loss of upper limb strength and function. Respiratory and heart failure occur, and patients finally require mechanical ventilator support to survive. The median life expectancy at birth is approximately 30 years. Currently, there is no treatment for DMD, and the standard of therapy is mostly focused on controlling disease symptoms and improving patient quality of life [15].

Nuclear factor kappa B (NF-kB) signaling pathway

Activation

**Figure 2:**
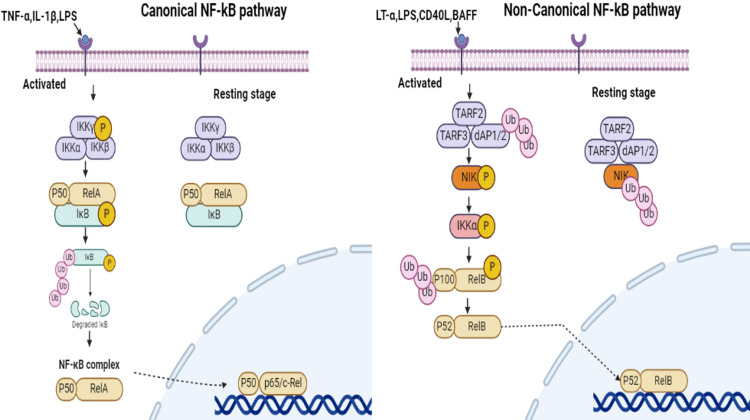
The figure contrasts two NF-κB signaling pathways related to immune response and inflammation. TNF-α, IL-1β, and LPS activate the canonical pathway, causing IκB degradation and NF-κB (p50/RelA) to enter the nucleus and activate genes. LT-α, CD40L, and BAFF activate a non-canonical pathway that involves NIK activation, p100 to p52 processing, and NF-κB (p52/RelB) entering the nucleus to regulate gene expression. Original illustration by the author.

This signaling system consists of five family members in mammals that form homodimers or heterodimers, i.e., RelA (p65), RELB, c-REL, NF-B1 (p105/p50), and NF-B2 (p100/p52) [16]. p65, RELB and c-REL have a part that binds to DNA and another part that turns on gene activity. In contrast, p50 and p52 only have the DNA-binding part and don’t have the part needed to turn on genes, so they can only help with starting gene activity but can't do it on their own [17]. The NF-κB signaling pathway is activated by a variety of stimuli, including cytokines, stress, free radicals, ultraviolet (UV) radiation, bacterial and viral antigens, antigen receptors, and damage-associated molecular patterns (DAMPs) [18]. A family of inducible transcription factors known as NF-κB controls various elements of inflammation and the immune system. NF-κB governs immune cell survival, activation, and differentiation in addition to promoting the expression of genes that are linked to inflammation [5]. These stimuli activate cell surface receptors like TNFR, IL-1R, and Toll-like receptors (TLRs), leading to the activation of kinases and adaptor proteins. DAMPs activate TLRs, activating the NF-κB pathway and contributing to DMD’s inflammatory response [8]. The IκB kinase complex (IKKα, IKKβ, and NF-κB essential modulator (NEMO)) is then activated [19]. This complex phosphorylates and degrades IκB proteins, releasing NF-κB dimers, which translocate to the nucleus and bind to specific DNA sequences to initiate gene transcription. The NF-κB signaling pathway can be activated through two distinct routes: the canonical (classical) pathway and the non-canonical (alternative) pathway (Figure 2). These pathways differ in their activation mechanisms, components, and biological outcomes. In the canonical pathway, activation of receptors like TLRs and cytokine receptors is necessary for canonical signaling because it attracts adaptor proteins like TRIF and MyD88 [3], activates IKKα and IKKβ, causing IκB breakdown and NF-κB (p65/p50) translocation to the nucleus to initiate gene transcription related to inflammation, immune response, and cell survival [17]. The non-canonical pathway requires NF-κB-inducing kinase (NIK) to convert p100 to p52, activating IKKα and phosphorylating the protein without IκB and the IKK complex [19]. Genes involved in the preservation of lymphoid tissue architecture and long-term immunological responses are regulated by p52/RelB dimers [2]. The canonical pathway is crucial for acute inflammatory responses, while the non-canonical pathway is essential for maintaining immune homeostasis and the development of secondary lymphoid organs. Regulatory mechanisms that control the NF-κB pathways are crucial for preventing chronic inflammation and disease. These mechanisms operate at multiple levels to ensure that NF-κB activity is appropriately modulated. Regulatory mechanisms, including inhibition by IκBs, ubiquitination, deubiquitinases (DUBs), negative feedback, and regulation of NIK and the IKK complex, ensure strict control of NF-κB pathways to prevent chronic inflammation, cancer, and autoimmune diseases [17]. For instance, IκBs bind to NF-κB dimers (like p65/p50) in the cytoplasm, preventing their translocation to the nucleus and keeping NF-κB inactive under non-stimulated conditions. The degradation of IκBs, which is required for NF-κB activation, is tightly regulated by ubiquitination and subsequent proteasomal degradation. Negative feedback mechanisms, such as the action of A20 and CYLD DUBs, help terminate NF-κB signaling by removing ubiquitin chains from key signaling molecules. Additionally, cross-talk with other signaling pathways and receptor desensitization also play roles in fine-tuning NF-κB activity. Non-coding RNAs, including microRNAs and long non-coding RNAs, further contribute to the regulation of NF-κB by targeting mRNAs of key signaling proteins or interacting with NF-κB regulators. These post-translational modifications and feedback mechanisms collectively ensure that NF-κB activity is tightly controlled to prevent excessive or prolonged activation, thereby reducing the risk of chronic inflammation, autoimmune disorders, and cancer.

Effect of NF- kB After Activation

The activation of NF-κB signaling exerts multifaceted effects across various biological processes, including inflammation, cell proliferation, cellular survival, and gene expression [20]. When NF-κB is activated, it undergoes translocation into the nucleus where it binds specifically to DNA sequences called κB sites. This interaction plays a crucial role in initiating the transcription of target genes involved in various cellular processes.

NF-κB in Inflammation

NF-κB activation initiates a cascade of gene expression involved in various inflammatory processes, notably through the induction of cytokines and chemokines. This includes increasing the synthesis of chemokines such as MCP-1 and MIP-1, along with cytokines like TNF-α, IL-1β, and IL-6, among others. These molecules serve as molecular signals that attract and activate immune cells at the site of inflammation, thereby intensifying and propagating the inflammatory response. Understanding these mechanisms is crucial for developing targeted therapies aimed at modulating NF-κB activity and managing inflammatory conditions effectively [21].

NF-κB in Innate Immune Cells

NF-κB plays a pivotal role in inflammation by regulating both innate and adaptive immune responses. In innate immunity, NF-κB supports critical processes such as the recruitment and survival of neutrophils, which are among the first responders to infections and tissue damage, essential for initiating a robust immune response. Moreover, NF-κB is involved in promoting the development and function of dendritic cells. Dendritic cells serve as crucial antigen-presenting cells that bridge the gap between innate and adaptive immunity, facilitating the activation of specific immune responses against pathogens. This multifaceted involvement underscores NF-κB’s significance in orchestrating immune responses across different phases of inflammation and immune defense, highlighting its potential as a therapeutic target for immune-related disorders [22].

NF-κB in Adaptive Immunity

NF-κB cells play a crucial role in the adaptive immune system by activating and isolating CD4 T-helper (Th) cells. When an antigen-presenting cell like a dendritic cell processes and presents an antigen, it interacts with naïve T cells through the T-cell receptor (TCR). This interaction is crucial for the initial activation of these naïve T cells, which then differentiate into various subsets of effector T cells, including CD4 Th cells. The NF-κB pathway is activated during this process, with RelA and c-Rel playing pivotal roles in transmitting signals from the TCR to the nucleus of the T cell. This signaling cascade leads to the expression of genes necessary for T cell activation, proliferation, and differentiation. However, if the NF-κB activation is not properly regulated, it can result in aberrant T-cell responses, contributing to the development of autoimmune diseases and chronic inflammation. Furthermore, NF-κB’s role extends beyond the initial activation of T cells; it also influences their subsequent isolation and the development of their effector functions, which are critical for a robust and effective adaptive immune response [23].

NF-κB in Cell Proliferation

Additionally, growth hormone (GH) and insulin-like growth factor I (IGF-I) boost NF-κB activity, which in turn promotes cell proliferation via the PI3K/Akt signal channel [24]. This activation leads to the induction of proliferative genes such as cyclin D1, which are pivotal for cell cycle progression and proliferation [25]. Growth factors that are known to encourage growth plate chondrogenesis and, thus, longitudinal bone development are growth factor-1 (IGF-1) and GH [24]. In murine pro-B lymphocytes, GH uses NF-kB to stimulate proliferation and inhibit apoptosis [26]. NF-κB has a part as a pro-inflammatory and anti-inflammatory regulator in mitochondrial-convinced inflammation. NF-κB anti-inflammatory functions help prevent excessive and inappropriate NLRP3-inflammasome activation, avoiding chronic inflammatory disease and inflammation caused by cell and tissue stress [27]. Proliferation, inflammation, and the function of immune cells are just a few of the cellular activities that NF-κB is an essential regulator of. Its activation results in the expression of various target genes that mediate these effects, highlighting its significance in both normal physiology and disease states.

NF-κB in DMD

In the context of DMD, NF-κB activation plays a significant role in muscle inflammation, fibrosis, and degeneration. DMD is characterized by chronic muscle inflammation, which is driven by the activation of NF-κB signaling pathways. This activation leads to the production of pro-inflammatory cytokines and chemokines that exacerbate muscle damage and inflammation. Additionally, NF-κB contributes to muscle fibrosis by promoting the expression of genes involved in extracellular matrix deposition, leading to increased fibrosis and impaired muscle regeneration. The chronic activation of NF-κB in DMD also affects muscle cell survival and function, ultimately contributing to muscle degeneration and the progressive loss of muscle mass and strength.

Role of NF-kb in DMD pathogenesis

Dysregulation of NF-kb Signaling in DMD

A significant factor contributing to the inflammatory processes observed in DMD is the aberrant regulation of NF-κB signaling. This dysregulation plays a pivotal role in initiating and sustaining inflammatory responses that contribute to the progressive deterioration of muscle tissue in individuals affected by DMD [28]. One important route in the inflammatory response in DMD is NF-κB signaling. The chronic inflammation, fibrosis, and muscle degeneration seen in DMD patients are caused by dysregulation of this system [2]. Degeneration and injury to muscles are facilitated by inflammation, a process driven by the persistent activation of NF-κB in muscle tissues. When muscles are damaged, they release molecules known as DAMPs. These DAMPs serve as signaling molecules that bind to TLRs, thereby triggering downstream signaling cascades. This sequence of events perpetuates inflammatory responses and contributes to the ongoing deterioration of muscle tissue seen in conditions like DMD [29]. Since TNF-α is produced and released by myofibers, it serves as a signaling molecule that communicates with satellite cells or myoblasts, impeding their normal development and differentiation processes. In the context of dystrophic conditions, the activation of NF-κB/IKK signaling pathways likely exacerbates this effect, further impairing muscle regeneration and contributing to the ongoing deterioration observed in muscular dystrophy [30].

Contribution of NF-κB to muscle inflammation, fibrosis, and degeneration

Inflammation

When NF-κB (Nuclear Factor kappa-light-chain-enhancer of activated B cells) is activated in DMD, it leads to the expression of numerous pro-inflammatory genes, resulting in ongoing inflammation and immune cell infiltration into muscle tissue. In DMD, the absence of dystrophin leads to continuous cycles of muscle damage and repair, further activating NF-κB in muscle cells and infiltrating immune cells. NF-κB also promotes the production of pro-inflammatory cytokines like TNF-α, IL-1β, and IL-6, amplifying the inflammatory response by attracting more immune cells to the site of damage. Additionally, NF-κB plays a key role in macrophage polarization toward the pro-inflammatory M1 phenotype, which further perpetuates tissue damage. This sustained inflammatory response exacerbates muscle injury and hinders the body’s natural repair processes, which are crucial for healing and regeneration. The dysregulation of NF-κB signaling is not only central to DMD but is also implicated in various inflammatory disorders. Mutations that affect NF-κB signaling can lead to conditions like immunodeficiency and autoinflammation across multiple organs, highlighting the broader impact of dysregulated NF-κB on inflammatory responses in muscular tissues. Furthermore, in DMD, NF-κB contributes to fibrosis by upregulating transforming growth factor-beta (TGF-β), replacing functional muscle tissue with fibrotic tissue and impairing muscle function. Chronic NF-κB activation also disrupts muscle regeneration by inhibiting the differentiation of muscle stem cells (satellite cells) into mature muscle fibers and inducing apoptosis in damaged muscle cells. This creates a vicious cycle of inflammation and degeneration, where ongoing NF-κB activity prevents effective muscle repair and regeneration, driving the progressive muscle-wasting characteristic of DMD [31]. Numerous inflammatory disorders can result from mutations that impact NF-κB signaling. For instance, immunodeficiency and autoinflammation across several organs are linked to abnormalities in NF-κB signaling brought on by particular genetic mutations. This also emphasizes the wider effects of dysregulated NF-κB on inflammatory responses in muscular tissues [18].

Fibrosis

Muscle cell NF-κB activation is associated with fibrosis and inflammation. By suppressing the expression of genes that are increased in muscle atrophy, such as MAFbx/Atrogin-1, Nedd4, and others, the production of a dominant negative IκBα in muscle cells lowers NF-κB activation and reduces muscle atrophy [17]. In DMD patients, NF-κB activation induces fibrotic factors like TGF-β, leading to excessive deposition of fibrotic tissue that replaces functional muscle tissue, impairing muscle function and complicating the repair and regeneration process. Additionally, NF-κB signaling plays a central role by promoting the expression of pro-inflammatory cytokines such as TNF-α, IL-1β, and IL-6, which create a pro-fibrotic environment by activating fibroblasts and enhancing extracellular matrix (ECM) production. Moreover, increased oxidative stress driven by NF-κB further exacerbates muscle damage and fibrosis, creating a vicious cycle that progressively impairs muscle function. TGF-β regulates the production of additional mediators and catabolic enzymes while stimulating the accumulation of extracellular matrix material. This contributes to fibrosis, replacing functional tissue with fibrotic scar tissue and impacting tissue function and repair mechanisms [32].

Degeneration

NF-κB signaling-induced inflammation directly damages muscle fibers and promotes fibrosis, impairing muscle function and complicating natural repair mechanisms [33]. In DMD patients, chronic NF-κB activation accelerates muscle fiber degeneration through increased protein degradation and inhibition of myogenic differentiation in satellite cells, crucial for muscle repair and regeneration. This persistent inflammation not only damages muscle fibers but also creates a fibrotic environment that further hampers the ability of satellite cells to regenerate muscle tissue. As a result, the progressive replacement of functional muscle with fibrotic tissue leads to a rapid decline in muscle function, exacerbating the overall disease progression and contributing to the severity of DMD [4].

Therapeutic strategies targeting NF-kB in DMD

Inflammatory disorders can be effectively managed through the targeted use of anti-inflammatory drugs that address multiple pathways concurrently. These medications typically aim to inhibit the NF-κB protein, which plays a central role in regulating DNA transcription, cytokine synthesis, and cell survival. NF-κB’s involvement spans diseases including cancer, arthritis, and inflammatory bowel disease, highlighting its significance in pathological inflammation. Understanding how anti-inflammatory drugs modulate NF-κB activation is essential for developing therapeutic approaches to mitigate inflammation-related conditions.

Current therapeutic strategies targeting NF-κB in DMD aim to inhibit this pathway to reduce inflammation, prevent muscle damage, and promote muscle regeneration. One approach involves using small molecule inhibitors that directly target NF-κB or its upstream regulators, thereby reducing the transcription of pro-inflammatory cytokines and other mediators of muscle degradation. Additionally, researchers are exploring gene therapy techniques to suppress NF-κB activity by modifying its signaling components or delivering microRNAs that can downregulate its expression. Other strategies include combining NF-κB inhibitors with other drugs to enhance overall treatment efficacy and reduce the inflammatory response more effectively.

Glucocorticoids, such as prednisone and deflazacort, are essential in treating DMD. They help manage inflammation, improve mobility, reduce muscle strength loss, enhance pulmonary function, and mitigate cardiomyopathy development, thereby potentially slowing disease progression and improving the quality of life for DMD patients [34]. These drugs work by inhibiting NF-κB signaling, which reduces the production of pro-inflammatory cytokines and other immune responses that contribute to muscle damage. By dampening the inflammatory response, glucocorticoids help slow the progression of muscle degeneration, improve muscle strength, and delay the onset of severe complications such as respiratory and cardiac failure.

However, the long-term use of glucocorticoids in DMD management is associated with significant side effects. These can include weight gain, osteoporosis, growth suppression, cataracts, hypertension, glucose intolerance, and increased risk of infections. Additionally, prolonged use can lead to muscle weakness, which paradoxically counteracts the intended benefits of the therapy. As a result, while glucocorticoids are effective in managing DMD symptoms, their long-term use requires careful monitoring and balancing of benefits against potential adverse effects.

Adeno-associated virus (AAV) vectors are preferred in gene therapy for DMD due to their ability to infect non-dividing cells and maintain prolonged gene expression. However, the large size of the *DMD* gene exceeds the capacity of AAV vectors, necessitating the use of smaller therapeutic genes, such as mini-dystrophin or micro-dystrophin. These engineered genes fit within the AAV payload capacity, enabling efficient delivery and expression of therapeutic sequences. Preclinical studies using animal models have shown promising results with these compact versions of dystrophin, reducing muscle pathology and enhancing function [35]. AAV vectors are a promising tool in gene therapy for DMD, a genetic disorder caused by mutations in the dystrophin gene. AAV vectors are utilized to deliver modified versions of the dystrophin gene, specifically mini-dystrophin or micro-dystrophin, into the muscle cells of patients. These truncated versions of the dystrophin gene are engineered to retain essential functional domains of the protein while being small enough to fit within the limited packaging capacity of AAV vectors, which is approximately 4.7 kilobases. Once inside the cells, the AAV-delivered gene allows for the production of a functional dystrophin protein, which can help stabilize muscle fibers and reduce the muscle degeneration that characterizes DMD.

However, delivering the *DMD* gene using AAV vectors presents several challenges. One significant issue is the immune response to both the AAV vector itself and the newly produced dystrophin protein, especially in patients who have developed antibodies due to previous exposure to the virus. This immune response can limit the effectiveness of the therapy or lead to adverse reactions. Another challenge is the large size of the dystrophin gene, which is one of the largest in the human genome, making it impossible to deliver the full-length gene using AAV vectors. This necessitates the use of mini-dystrophin or micro-dystrophin, which, while functional, are not as effective as the full-length protein. Additionally, achieving widespread and sustained expression of the gene throughout all affected muscles, including the heart and diaphragm, remains difficult due to the limited ability of AAV vectors to reach and transduce all muscle tissues efficiently.

To address these challenges, researchers are developing strategies to suppress the immune response, such as using immunosuppressive drugs or designing AAV vectors that are less likely to be recognized by the immune system. Advances in vector engineering are also being made to enhance the targeting and transduction efficiency of AAV vectors. Preclinical studies using animal models have shown promising results with these compact versions of dystrophin, reducing muscle pathology and enhancing function [35]. Preclinical studies in animal models have shown that mini-dystrophin and micro-dystrophin can partially restore muscle function and significantly reduce muscle degeneration, indicating that these truncated versions of dystrophin can be therapeutically effective in DMD. Ongoing clinical trials are investigating the safety and efficacy of these gene therapies in humans, with early results showing promise in terms of improving muscle function and slowing disease progression.

Patient perspectives and supportive care

Insights from Patients and Caregivers on Living with DMD

Children with DMD are often asymptomatic in their early years, gradually developing muscle weakness and typically losing the ability to walk between ages 8 and 10 [36]. DMD presents a host of challenges for both patients and their caregivers, significantly impacting their quality of life. The progressive nature of the disease, which leads to severe muscle weakness and loss of mobility, means that individuals with DMD gradually lose the ability to perform basic tasks such as walking, dressing, and eating independently. Symptoms include fatigue, muscle weakness starting in the pelvis and legs and spreading to the neck and arms, and learning impairments. Motor skill impairment leads to frequent falls, difficulty climbing stairs, missed motor milestones, loss of ambulation, and serious cardiac and pulmonary issues [37]. This loss of independence is often accompanied by the need for assistive devices, such as wheelchairs, and eventually respiratory support as the disease progresses to affect respiratory and cardiac muscles. The physical limitations imposed by DMD are often coupled with chronic pain, fatigue, and frequent respiratory infections, further diminishing the quality of life.

For caregivers, the challenges are both emotional and physical. They must manage the complex needs of the patient, which can include administering medications, assisting with daily activities, and coordinating medical care, often requiring them to make significant personal sacrifices. The emotional burden of watching a loved one’s health deteriorate can lead to stress, anxiety, and depression among caregivers. The constant care required can also lead to physical exhaustion and, in many cases, financial strain due to the cost of treatment, specialized equipment, and potential loss of income if the caregiver must reduce work hours or leave their job to provide full-time care. The standard medical treatments for DMD, like glucocorticoids, can cause significant side effects, including growth limitations, weight gain, anxiety, moon face, mental imbalance, and osteoporosis [38].

The symptoms and progression of DMD affect every aspect of life for the patient, including education, social interactions, and psychological well-being. Children with DMD may face difficulties in school due to cognitive impairments associated with the disease and the physical limitations that make participation in many activities challenging. As the disease progresses, social isolation becomes a significant issue, as the physical limitations and the need for special accommodations can make it difficult to engage in social activities. This isolation can lead to feelings of loneliness and depression, further affecting the individual’s mental health. Overall, the progression of DMD leads to a gradual erosion of quality of life, creating a profound impact on both the patient and their caregivers.

Important measures in managing the disease

Respiratory Management

Maintaining optimal lung health is critically important for individuals with DMD. To support this, it is essential for patients to receive annual vaccinations for both influenza and pneumonia. In the early stages of the disease, lung examinations are typically conducted based on the presence of symptoms. However, as the condition progresses and the patient becomes less mobile, these examinations should be performed every six months to closely monitor respiratory function. Additionally, the use of idebenone has been shown to help delay the onset of respiratory complications, offering a beneficial intervention in the management of lung health in DMD patients [39]. Managing respiratory health in DMD patients is a critical aspect of supportive care, as respiratory complications are a leading cause of morbidity and mortality in this population. As the disease progresses, respiratory muscles weaken, leading to reduced lung function, hypoventilation, and an increased risk of respiratory infections. An anticipatory strategy for respiratory care involves implementing techniques such as lung volume recruitment, cough assistance, nocturnal ventilation support, and timely daytime ventilation, alongside continuous monitoring of respiratory muscle function. These proactive measures aim to optimize respiratory health and manage potential complications effectively in advance [40]. Essential supportive care measures include regular monitoring of respiratory function through pulmonary function tests, such as forced vital capacity (FVC) and peak cough flow, to assess the need for interventions.

Non-invasive ventilation (NIV) is commonly used to support breathing, especially during sleep when hypoventilation typically worsens. NIV helps maintain adequate oxygen levels, removes carbon dioxide, and reduces the work of breathing, which can significantly improve quality of life and survival rates. For patients with more advanced disease, continuous NIV or invasive ventilation via tracheostomy may be required.

Airway clearance techniques are also crucial to manage respiratory secretions and prevent infections. This includes methods such as chest physiotherapy, mechanical insufflation-exsufflation devices (cough assist), and regular use of nebulized saline or mucolytics to thin secretions. Additionally, the use of idebenone has been shown to help delay the onset of respiratory complications, offering a beneficial intervention in the management of lung health in DMD patients [39]. Additionally, ensuring timely vaccination against respiratory pathogens like influenza and pneumococcus is important to prevent infections that could exacerbate respiratory decline.

In some cases, supplemental oxygen may be needed, but it is used cautiously, as it can suppress respiratory drive in patients with chronic CO_2_ retention. Comprehensive respiratory care also involves educating caregivers and patients about the signs of respiratory distress and ensuring access to emergency care plans for respiratory crises. This holistic approach, combining monitoring, ventilation support, airway clearance, and infection prevention, is essential in managing respiratory health in DMD patients.

Gastrointestinal and Nutritional Management

People with DMD may encounter challenges concerning their gastrointestinal system and nutritional needs. These issues can manifest as fluctuations in weight, reduced bone density, swallowing difficulties, and elevated potassium levels (hyperkalemia) [41]. Nutritional management plays a crucial role in improving the health and quality of life of individuals with DMD by addressing specific dietary needs and mitigating complications associated with the disease. Due to progressive muscle weakness and reduced physical activity, individuals with DMD often experience a decline in muscle mass and an increased risk of obesity, which can exacerbate mobility issues and metabolic problems. A well-balanced diet tailored to the patient’s needs can help manage body weight, prevent nutritional deficiencies, and support overall health. Key elements of nutritional management include providing adequate caloric intake to maintain energy levels and support muscle mass while avoiding excessive weight gain. This often involves a diet rich in lean proteins, healthy fats, and complex carbohydrates to support muscle function and repair.

When a multidisciplinary team comprising experts like gastroenterologists, swallowing therapists, and nutritionists delivers comprehensive nutritional care, it can significantly enhance the quality of life for individuals with DMD. Moreover, patients with DMD are at risk of gastrointestinal issues, such as constipation and dysphagia (difficulty swallowing), which can be managed through appropriate dietary adjustments. Incorporating high-fiber foods can alleviate constipation while modifying food textures and using thickeners can help address swallowing difficulties. Additionally, ensuring adequate intake of vitamins and minerals, particularly calcium and vitamin D, is essential to support bone health and counteract the risk of osteoporosis, a common side effect of long-term glucocorticoid use in DMD patients.

With advancements in therapy contributing to increased life expectancy among DMD patients, there is a growing need to address nutritional aspects and potential long-term impacts as they transition into adulthood. This underscores the importance of proactive management and tailored nutritional interventions in improving overall health outcomes [42]. As the disease progresses, there is typically a transition from overnutrition to undernutrition [43]. As the illness progresses, many individuals experience increasing challenges with swallowing and eating. Oral-pharyngeal dysphagia often begins with subtle symptoms that can develop gradually, leading patients to potentially underestimate or underreport its impact [44]. The primary objective of nutritional management is to effectively monitor weight and growth, alongside providing a well-balanced diet. This approach aims to prevent the onset of obesity, undernutrition, and malnutrition, ensuring overall health and wellness for individuals undergoing care [45].

Emergency Management

A crucial component of continuous therapeutic support is educating patients and their caregivers about identifying emergencies and understanding appropriate actions to take in such situations. Equipping patients with personalized documents containing essential medical information during emergencies not only helps mitigate risks but also plays a vital role in improving overall patient outcomes and ensuring timely and effective intervention [46]. Emergency management strategies for DMD patients are crucial due to the potential for severe complications related to respiratory and cardiac systems, as well as other acute issues arising from the disease. Critical strategies include establishing a comprehensive emergency plan that covers common scenarios, such as respiratory distress, cardiac events, and sudden health deterioration. For respiratory emergencies, caregivers should be trained in the use of NIV devices, like bilevel positive airway pressure (BiPAP), and mechanical insufflation-exsufflation devices to assist with breathing and clear secretions. They should also recognize signs of respiratory distress, such as increased work of breathing, changes in oxygen saturation, and difficulty speaking or maintaining alertness.

In the case of cardiac emergencies, caregivers need to be aware of symptoms indicative of heart problems, such as chest pain, palpitations, or sudden changes in breathing patterns. Familiarity with emergency protocols, including the use of automated external defibrillators (AEDs) and administering cardiopulmonary resuscitation (CPR), is essential. Regular monitoring and management of potential complications like hyperkalemia or hypoglycemia, often associated with glucocorticoid treatment, should also be part of the care plan.

To optimize patient outcomes, it is essential that medical providers must possess a thorough understanding of the unique challenges associated with managing emergencies for individuals diagnosed with DMD. This specialized knowledge enables healthcare teams to implement targeted and effective emergency response strategies tailored to the specific needs of DMD patients, thereby improving overall care and outcomes in critical situations [47]. Education for caregivers should include practical training sessions on the use of medical equipment, recognition of critical symptoms, and emergency response techniques. Caregivers should also be educated on how to access emergency medical services quickly, how to communicate the patient's specific needs and history effectively, and how to maintain a prepared emergency kit that includes essential medications, equipment, and contact information. This education ensures that caregivers can respond promptly and effectively to emergencies, improving outcomes and reducing the risk of severe complications for DMD patients.

## Conclusions

Dystrophin, a vital protein that stabilizes muscle fibers during contraction, is absent in young males with DMD, a debilitating and degenerative hereditary condition. The *DMD* gene is mutated to cause this absence; these mutations frequently involve substantial deletions, duplications, or point mutations that alter the reading frame of the gene. Muscle fibers that lack dystrophin are brittle and more likely to sustain damage from routine tasks, which can result in recurrent cycles of inflammation, muscle cell death, and fibrosis. The NF-κB signaling pathway plays a major role in mediating the chronic inflammatory response. In DMD, this system is constantly active due to ongoing muscle injury and stress. The expression of pro-inflammatory cytokines, chemokines, and adhesion molecules is driven by NF-κB, which is normally involved in regulating immune responses. This results in the persistence of chronic inflammation inside the affected muscles. Prolonged inflammation draws immune cells such as T cells and macrophages, which unintentionally worsen muscle degradation and encourage the replacement of muscle tissue with fibrous scar tissue in an effort to repair damage. Muscle function is severely compromised by this fibrotic process, which also adds to the gradual muscle weakening that characterizes DMD. Furthermore, because NF-κB plays a role in muscle pathology, treatments that target this pathway may be able to reduce fibrotic and inflammatory processes, reducing the advancement of the disease and possibly maintaining muscle function. The current focus of treatment is on symptom management. Physical therapy is used to preserve mobility, corticosteroids are used to reduce inflammation, and ventilatory assistance is provided when breathing difficulties arise. On the other hand, more direct interventions are being investigated by developing therapies, including gene therapy to restore dystrophin expression, exon-skipping tactics to avoid mutations, and innovative anti-inflammatory medications to lessen the negative consequences of NF-κB activation. There is potential for changing the trajectory of DMD with these novel medicines.
